# Transgenic iPSC Lines with Genetically Encoded MitoTimer to Study Mitochondrial Biogenesis in Dopaminergic Neurons with Tauopathy

**DOI:** 10.3390/biomedicines13030550

**Published:** 2025-02-21

**Authors:** Julia A. Nadtochy, Sergey P. Medvedev, Elena V. Grigor’eva, Sophia V. Pavlova, Julia M. Minina, Anton V. Chechushkov, Anastasia A. Malakhova, Liudmila V. Kovalenko, Suren M. Zakian

**Affiliations:** 1 Institute of Cytology and Genetics, Siberian Branch of Russian Academy of Sciences, Novosibirsk 630090, Russia; y.nadtochii@g.nsu.ru (J.A.N.); medvedev@bionet.nsc.ru (S.P.M.); evlena@bionet.nsc.ru (E.V.G.); spav@bionet.nsc.ru (S.V.P.); minina_jul@bionet.nsc.ru (J.M.M.); zakian@bionet.nsc.ru (S.M.Z.); 2 Department of Natural Sciences, Novosibirsk State University, Novosibirsk 630090, Russia; 3 Institute of Chemical Biology and Fundamental Medicine, Siberian Branch of Russian Academy of Sciences, Novosibirsk 630090, Russia; achechushkov@gmail.com; 4 Federal Research Center of Fundamental and Translational Medicine Siberian Branch of Russian Academy of Sciences, Novosibirsk 630090, Russia; 5 Department of Pathophysiology and General Pathology, Medical Institute, Khanty-Mansiysk Autonomous Okrug–Ugra Surgut State University, Surgut 628403, Russia; kovalenko_lv@surgu.ru

**Keywords:** mitochondrial dysfunction, MitoTimer biosensor, induced pluripotent stem cells, CRISPR/Cas9, *MAPT:c.2013T > G*, frontotemporal dementia with parkinsonism-17

## Abstract

**Background:** Tauopathy has been identified as a prevalent causative agent of neurodegenerative diseases, including frontotemporal dementia with parkinsonism-17 (FTDP-17). This rare hereditary neurodegenerative condition is characterised by the manifestation of parkinsonism and behavioural changes. The majority of cases of FTDP-17 are associated with mutations in the *MAPT* gene, which encodes the tau protein. *MAPT* mutations lead to disruption of the balance between 3R and 4R tau forms, which causes destabilisation of microtubules and impairment of cellular organelle functions, particularly mitochondrial dysfunction. The development of model systems and tools for studying the molecular, genetic, and biochemical mechanisms underlying FTDP-17 and testing therapies at the cellular level is an urgent necessity. **Methods:** In this study, we generated transgenic lines of induced pluripotent stem cells (iPSCs) from a patient carrying the pathogenic mutation c.2013T > G (rs63750756, p.N279K) of *MAPT* and a healthy donor. A doxycycline-controlled transgene of the genetically encoded biosensor MitoTimer was integrated into the *AAVS1* locus of these cells. The MitoTimer biosensor allows for lifetime monitoring of the turnover of mitochondria in neuronal cells derived from directed iPSC differentiation. The fact that transcription of the transgene can be induced by doxycycline provides additional possibilities for pulse labelling of newly formed mitochondria. **Results:** Transgenic iPSC lines provide a unique tool to study the molecular and genetic mechanisms of FTDP-17 caused by the presence of the c.2013T > G (p.N279K) mutation, as well as to test potential drugs in vitro.

## 1. Introduction

Tauopathies are a broad group of neurodegenerative diseases of the central nervous system characterised by abnormal accumulation of fibrillar aggregates of tau protein in brain tissue. This group includes diseases that vary in phenotypic manifestation and are hereditary or sporadic in nature, including Alzheimer’s disease, primary age-related tauopathy, Pick’s disease, progressive supranuclear palsy, frontotemporal dementia, and others [1]. Recent evidence suggests that the tau protein encoded by the *MAPT* (*Microtubule Associated Protein Tau*; OMIM *157140) gene located on chromosome 17 may play a central role, independent of alpha-synuclein, in the degeneration of the substantia nigra of the brain and in the development of parkinsonism and Parkinson’s disease [2,3]. To date, more than fifty genetic variants in the *MAPT* gene have been identified, a significant proportion of which have been associated with the development of neurodegenerative diseases and have been shown to affect tau aggregation, the ratio of its isoforms (3R and 4R), tau binding to microtubules, and involvement in their assembly [4,5,6,7]. The mutation *MAPT:c.2013T > G* (rs63750756) has been shown to be a causative factor in the development of frontotemporal dementia with parkinsonism-17 (FTDP-17; OMIM # 600274), a condition that is characterised by tauopathy.

The substitution c.2013T > G in *MAPT* is classified as a missense mutation, the primary effects of which are manifested at the level of mRNA [5]. It has been established that this mutation leads to an augmentation of the polypurine-positive cis element present in exon 10. This has been demonstrated to enhance the frequency at which exon 10 is incorporated into the transcript during the splicing process and to modify the ratio of tau protein isoforms 3R and 4R, with a tendency towards a decrease [8].

The altered ratio of 3R and 4R forms results in the destabilisation of microtubules and the disruption of subcellular vesicle transport [7,9,10,11,12]. Furthermore, an increased incidence of calcium fluctuations was observed in *MAPT:c.2013T > G* neurons, resulting in hyperpolarisation and division of mitochondria. This, in turn, resulted in an escalation in the generation of reactive oxygen species within the mitochondria, which further contributed to the onset of cell death [9]. For a more detailed study of the relationship between the tau protein and mitochondrial dysfunction, it is necessary to create new model systems and tools. Such tools may include patient-specific iPSCs with integrated biosensor systems [13].

The MitoTimer ratiometric biosensor has been developed as a tool to study mitochondrial biogenesis [14,15,16,17]. The MitoTimer biosensor involves the green fluorescent Timer protein, which is localised in the mitochondria. The Timer protein has been fused with the COX8A subunit guide sequence, which facilitates the transfer of the MitoTimer to the mitochondrial matrix. Immediately following translation and folding, the Timer exhibits green fluorescence, and over time, the emission peak undergoes a shift to red. The observed MitoTimer colour is determined by the duration of Timer expression and the rate of incorporation and degradation of the mitochondrial protein [14,17]. The ratio of the red/green signal can be utilised to track mitochondrial biogenesis, and an increase in this ratio is indicative of an accumulation of old protein, potentially attributable to impaired mitophagy or increased mitochondrial stress. Conversely, a decrease in the red/green ratio can be indicative of a predominance of newly synthesised protein or may be attributable to increased mitophagy [16].

The MitoTimer biosensor has previously been shown to be applicable to studying changes in mitochondrial structure, mitophagy, and oxidative stress in a wide range of models, including the transgenic nematode *Caenorhabditis elegans*, the fly *Drosophila melanogaster*, and mice, as well as primary cell cultures and various stable cell lines. It has been used to study mitochondrial function in skeletal muscle, heart, liver, and hippocampal neuronal cells [15,17,18,19,20,21]. In addition, using the insulin-producing rat insulinoma cell line INS1 as an example, MitoTimer was shown to be successful in screening libraries of thousands of small molecules to find compounds that stimulate basal mitophagy, which positively affects mitochondrial function, activity, and expression of Complex I, Complex II, and Complex III, which may mediate the improvement in insulin secretion in mouse pancreatic islets [22]. Although the MitoTimer is a working tool for studying mitochondria in live cells and in real time, and there is ample evidence that tau protein dysfunction significantly affects mitochondrial function [23,24], we found no published studies using this biosensor to generate and study human neurodegenerative disease models based on differentiated iPSC derivatives in general and frontotemporal dementia with parkinsonism-17 in particular.

The obtained transgenic iPSCs express the MitoTimer protein, controlled by a doxycycline-inducible promoter. These iPSCs can serve as a cellular model to study the mechanisms of disease development and can also be used to screen potential drugs. The present study generated and characterised two transgenic iPSC lines of a patient with FTDP-17 with a pathological genetic variant of *MAPT:c.2013T > G* (rs63750756, p.N279K), as well as two transgenic lines of a healthy donor.

## 2. Materials and Methods

### 2.1. Cell Lines

The study utilised iPSCs derived from a patient with FTDP-17 caused by the pathogenic genetic variant *MAPT:c.2013T > G* PD57-6 (ICGi052-A) and PD57-7 (ICGi052-B) (https://hpscreg.eu/cell-line/ICGi052-B, last accessed 28 December 2024). Additionally, iPSCs derived from a healthy donor K7-4Lf (ICGi022-A) (https://hpscreg.eu/cell-line/ICGi022-A, last accessed 28 December 2024) were used as a control. The generation and detailed characterisation of these cell lines are described in [25,26].

### 2.2. Cultivation of iPSCs

iPSCs were cultured on a feeder layer of mouse embryonic fibroblasts treated with mitomycin C (Sigma-Aldrich, Darmstadt, Germany) according to previously described methods [25,26].

### 2.3. Derivation of Transgenic iPSCs

The pAAVS1-TRE-mCMV-MitoTimer genetic construct was obtained by amplifying a fragment containing the MitoTimer sequence and the SV40 polyadenylation signal using the primers MitoTimer-MluI-F 5′-GTCACGCGTGCCACCATGTCCGCCTGACGCCGTG-3′ and MitoTimer-AflII-R 5′-CGCCTTAAGATACATTGATGAGTTTG-3′ and pMitoTimer plasmids (Addgene plasmid #52659; http://n2t.net/addgene:52659, last accessed 28 December 2024; RRID: Addgene_52659) [17] as a matrix, followed by cloning of the MluI-AflII fragment in the pCyto-GFP2-Orp1-donor vector [27]. Transfection of iPSCs was performed on a Neon Transfer System (Thermo Fisher Scientific, Waltham, MA, USA) according to the manufacturer’s instructions, using a programme: 1100 V, 30 ms, 1 pulse. For a 100 µL transfection reaction, 4–5 × 10^5^ cells and 1 µg of each of the following plasmids were used: pX458-AAVS1 encoding SpCas9 nuclease, EGFP, and *AAVS1* sgRNA (based on pSpCas9(BB)-2A-GFP (Addgene #48138; http://n2t.net/addgene:48138, last accessed 28 December 2024; RRID: Addgene_48138) [28]; the donor plasmid AAVS1-Neo-M2rtTA, encoding a reverse transactivator for doxycycline-controlled expression and a neomycin resistance gene (Addgene plasmid #60843; http://n2t.net/addgene:60843, last accessed 28 December 2024; RRID: Addgene_60843) [29] and the donor plasmid pAAVS1-TRE-mCMV-MitoTimer.

Following electroporation, the cells were seeded onto a feeder layer in an antibiotic-free iPSC culture medium, with the addition of 2 µM of thiazovivin. The cells were cultivated at 37 °C in a CO_2_ incubator in a humid atmosphere containing 5% carbon dioxide. Forty-eight hours post-transfection, 50 µg/mL of geneticin (G418) was added to a penicillin–streptomycin-free culture medium for a period of four to five days. Following the removal of G418, a 24-h period was allowed to elapse before the cells were selected for resistance to 250 ng/mL puromycin (Santa Cruz Biotechnology, Dallas, TX, USA) for a further 3–4 days. Thereafter, surviving colonies were mechanically plated into 48-well plates. The integration of the transgenes was analysed by PCR (primers are listed in Table 1) as previously described [27].

### 2.4. Karyotyping

As previously outlined [30], the procedure of the karyotype analysis comprised the following steps. Initially, cells were treated with ethidium bromide (3 μg/mL) and colcemid (50 ng/mL) (BioloT, Saint Petersburg, Russia) for 2.5 h. Thereafter, the cells were subjected to a hypotonic solution (0.075 M KCl) for 20 min at a temperature of 37 °C. The cells were then fixed in ice-cold fixative (three parts methanol, one part glacial acetic acid) for a period of 15 min. The suspension was then dropped onto wet, cooled slides. The analysis of chromosomes was conducted using an Axioplan 2 microscope (Zeiss, Oberkochen, Germany) within the Common Facilities Center of Microscopic Analysis of Biological Objects, ICG SB RAS (https://ckp.icgen.ru/ckpmabo/, last accessed 28 December 2024), which is supported by the state project of the Institute of Cytology and Genetics (FWNR-2022-0015).

### 2.5. Spontaneous Differentiation of iPSCs in Embryoid Bodies

The spontaneous differentiation of iPSCs was achieved by the formation of embryoid bodies, as previously described [31]. In summary, the cells were detached from the substrate using 0.15% collagenase IV (Thermo Fisher Scientific, Waltham, MA, USA) and then seeded onto dishes coated with 1% agarose in iPSC medium without bFGF for a period of 10–14 days. The resulting spheroids were transferred to Matrigel-treated 8-well coverglass imaging plates (Thermo Fisher Scientific, Waltham, MA, USA) and cultured for a further 7–10 days before immunofluorescence analysis was performed.

### 2.6. Immunofluorescence Analysis

The immunofluorescence staining procedure was conducted as follows: the cells were first fixed with 4% paraformaldehyde (PFA) (Sigma-Aldrich, Darmstadt, Germany), then permeabilised with 0.5% Triton X-100 (Thermo Fisher Scientific, Waltham, MA, USA) and subsequently incubated with 1% bovine serum albumin (BSA) (Sigma-Aldrich, Darmstadt, Germany) for a duration of 30 min. Primary antibodies were then added for an overnight incubation at +4 °C, after which secondary antibodies were applied for 1.5 h at room temperature, as outlined in [31]. The nuclei were then contrasted with DAPI. The complete list of antibodies used can be found in Table S1.

### 2.7. Directed Differentiation into Midbrain Neuron Derivatives

The dopaminergic neurons were derived from iPSCs in accordance with a previously published protocol [31], with modifications as outlined in [32]. iPSCs were seeded onto Matrigel-GFR extracellular matrix-coated plates (Corning, New York, NY, USA) in a feeder-free condition and cultured in Essential 8 Medium Kit (Thermo Fisher Scientific, Waltham, MA, USA) to a density of 80–90% for 24 h. Thereafter, the iPSC culture medium was replaced with a neuronal differentiation medium comprising F12/DMEM:Neurobasal (1:1), 0.5× N-2, and 0.5× B-27 supplements without vitamin A, 0.2 mM GlutaMAX™, 100 U/mL penicillin–streptomycin (all from Thermo Fisher Scientific, Waltham, MA, USA), and 200 μM ascorbic acid (Sigma-Aldrich, Darmstadt, Germany). The factors were then added as described in [32]. For cell passaging at days 11, 18, and 25 of differentiation, the StemPro Accutase (Thermo Fisher Scientific, Waltham, MA, USA) was utilised. The cells were then seeded at a 1:2 ratio onto Matrigel-coated plates in differentiation medium containing 2 µM of thiazovivin.

In order to investigate MitoTimer readouts in neuronal cultures, the MitoTimer biosensor was activated by the addition of 2 μg/mL of doxycycline (Sigma-Aldrich, Darmstadt, Germany) to the culture medium. To estimate the influence of antioxidants on mitochondrial turnover, the cells were cultured for 48 h in an antioxidant-depleted medium containing F12/DMEM:Neurobasal (1:1), 0.5× N-2, and 0.2 mM GlutaMAX™, 100 U/mL penicillin–streptomycin (all from Thermo Fisher Scientific, Waltham, MA, USA) with the addition of 2 μg/mL of doxycycline. Images were captured using a Nikon Eclipse Ti-E microscope (Nikon, Tokyo, Japan) and NIS Elements Advanced Research software version 4.30.

### 2.8. DNA and RNA Isolation

The QuickExtract™ DNA extraction solution (Lucigen, Madison, WI, USA) was utilised for the isolation of genomic DNA from iPSCs.

For RNA isolation, iPSCs or derivatives of midbrain neurons cultivated in a 35 mm diameter Petri dish were collected at days 55–60 of differentiation. The cells were lysed in 1 mL of TRIzol (Ambion by Life Technologies, Carlsbad, CA, USA), and RNA was isolated in accordance with the manufacturer’s protocol. The reverse transcription reaction was then performed on 1 µg of total RNA using M-MuLV-RH reverse transcriptase (Biolabmix, Novosibirsk, Russia).

### 2.9. Qualitative and Quantitative PCR

PCR was performed using the BioMaster HS-Taq PCR-Color Kit (2×) (Biolabmix, Novosibirsk, Russia) on a T100 Thermal Cycler (Bio-Rad Laboratories, Singapore). The programme for the detection of transgenes, wild-type *AAVS1* alleles, and non-target inserts is as follows: 95 °C for 5 min; 35 cycles of 95 °C for 30 s, 62–64 °C for 30 s, 72 °C for 30 s; 72 °C for 5 min. The programme for the detection of Mycoplasma contamination is as follows: initial heating to 95 °C for 5 min, followed by 35 cycles of 15 s at 95 °C, 15 s at 60 °C and 20 s at 72 °C, and finally 5 min at 72 °C. The primer sequences can be found in Table 1.

Quantitative PCR (qPCR) was performed on a LightCycler 480 II system (Roche, Switzerland) according to the following programme: Initial denaturation at 95 °C for 5 min, followed by 40 cycles of denaturation at 95 °C for 15 s and annealing/extension at 62 °C for 45 s. The BioMaster HS-qPCR SYBR Blue 2× Kit (Biolabmix, Novosibirsk, Russia) was used. Pluripotency marker expression results were normalised to beta-2-microglobulin (*B2M*). The HUES9 embryonic stem cells [33] were used as a reference line. The results were processed using the ∆∆CT method [34] using qbase+ V3.4.

### 2.10. Sanger Sequencing

To confirm the presence of a mutation in the *MAPT* gene in iPSCs of patients with FTDP-17, Sanger sequencing was performed using the BigDye Terminator V Cycle Sequencing Kit 3.1. (Applied Biosystems, Austin, TX, USA). The primer sequences are provided in Table 1. For the reaction, 0.5 μL BigDye™ Terminator Version 3.1 was mixed with 1.75 μL of 5x sequencing buffer (containing 250 mM of Tris-HCl (pH 9 at 25 °C) and 10 mM of MgCl_2_), 1 μL of 10 μM primer, and 30–50 ng of template DNA (a purified PCR product), and deionised water to a final volume of 10 μL. The sequencing reactions were carried out on a T100 Thermal Cycler (Bio-Rad Laboratories, Singapore) using a program of 96 °C for 5 min, followed by 50 cycles of 96 °C for 20 s, 56 °C for 15 s, and 60 °C for 4 min. The results were analysed using an ABI 3130XL genetic analyser at the SB RAS Genomics Core Facility (http://www.niboch.nsc.ru/doku.php/corefacility, last accessed 28 December 2024).

### 2.11. Calculation of Red/Green Fluorescence Ratio of the MitoTimer Biosensor

Images of MitoTimer fluorescence were captured using an LSM 710 confocal microscope (Zeiss, Oberkochen, Germany) and ZEN Black 2009 software. LSM710 images were acquired with the following settings: objective alpha Plan-Apochromat 100×/1.46 Oil DIC, excitation lasers 488 nm Argon (max 250 mW) and 543 nm HeNe (max power 1.2 mW) were set to 2% of their maximum, respectively, with dichroic filters 488 nm and 458/543 nm; emission signal channel bandpass were set as follows: 493–530 nm (Green channel) and 548–611 nm (Red channel); and pixel dwell was set to 0.64 microseconds per pixel for each channel. Images were acquired in Z-stack modes. For red/green ratio calculation, images were processed using ImageJ 1.54f software. Statistical processing was performed using the ANOVA test to determine significant differences between groups.

## 3. Results

### 3.1. Detection of 3R and 4R Forms of MAPT in iPSC-Derived Dopaminergic Neurons

**Figure 1:**
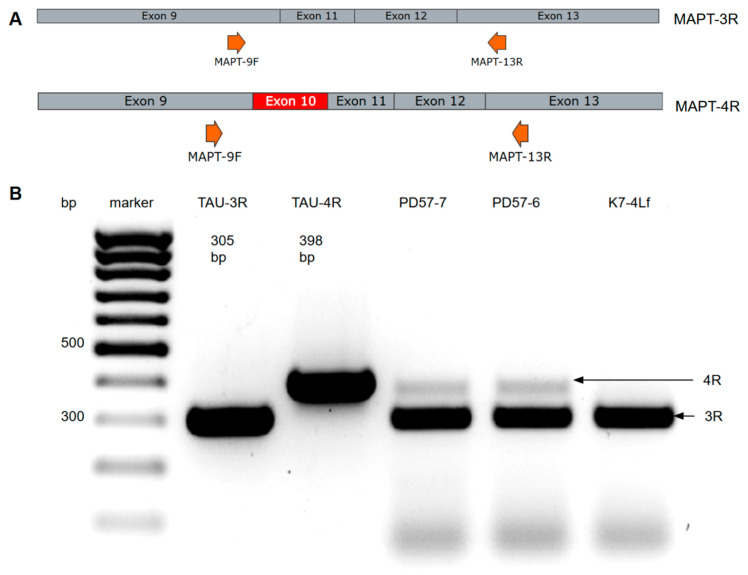
PCR analysis of the expression of the 3R and 4R forms of the *MAPT* mRNA in PD57-7, PD57-6, and K7-4Lf iPSC-derived neurons. (**A**) Arrangement of primers for RT-PCR. The arrows indicate the direction of the primers. In the case of the 3R form, PCR yields the 305 bp product. The 4R mRNA form containing exon 10 gives a 398 bp PCR product. (**B**) Analysis of the expression of the mRNA forms of the *MAPT* gene in the iPSC-derived DA neurons by RT-PCR. Arrows indicate bands interpreted as 3R and 4R forms of *MAPT* gene mRNA. Samples of iPSC-derived neurons from the FTDP-17 (PD-57) patient show the presence of PCR products corresponding to the 4R form. Plasmids containing 3R and 4R cDNA forms of the *MAPT* mRNA gene (TAU-3R and TAU-4R) were used as controls.

The *MAPT:c.2013T > G* genetic variant is a 4R tauopathy, suggesting increased expression of the 4R form of the protein. To evaluate the expression of the *MAPT* mRNA forms with or without exon 10 (producing the 4R and 3R forms of tau protein, respectively) in the dopaminergic neurons derived from iPSC lines PD57-7 (ICGi052-B), PD57-6 (ICGi052-A), K7-4Lf (ICGi022-A) [25,26], we performed qualitative PCR with cDNA using MAPT-9F and MAPT-13R primers (Figure 1A).

As anticipated, the electropherogram revealed the presence of a band corresponding to the 4R form in the patient’s cells, as demonstrated in Figure 1B. The experiment was conducted using plasmids containing cDNA of mRNA forms of the *MAPT* gene (TAU-3R and TAU-4R) to serve as controls.

### 3.2. Generation and Characterisation of Transgenic iPSC Lines Carrying the MitoTimer Biosensor

**Figure 2:**
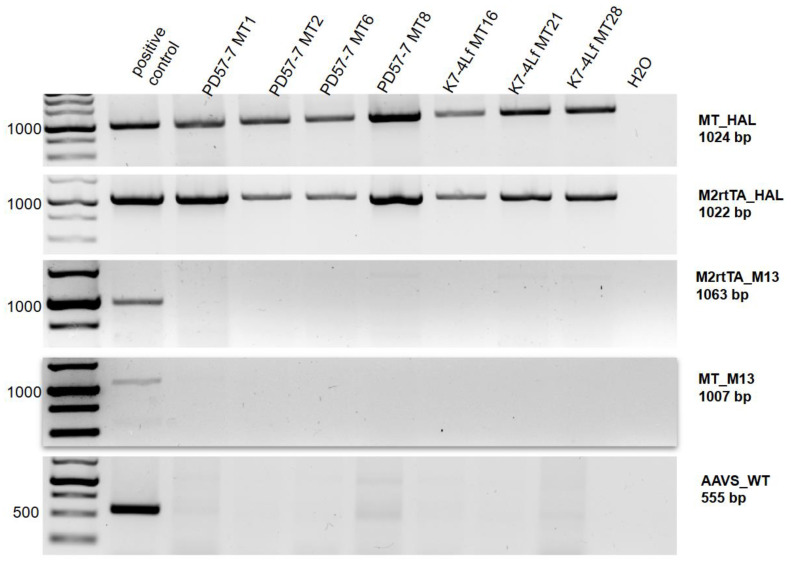
PCR analysis to verify the successful integration of the MitoTimer biosensor and doxycycline-dependent transactivator transgenes into the *AAVS1* locus. MT_HAL: screening for integration of the MitoTimer biosensor transgene into the *AAVS1* locus, M2rtTA_HAL: screening for integration of the M2rtTA transgene into the *AAVS1* locus, MT_M13: screening for off-target insertion of the MitoTimer donor plasmid into the genome, M2rtTA_M13: screening for off-target insertion of the AAVS1-NEO-M2rtTA donor plasmid into the genome, AAVS_wt: screening against the wild-type *AAVS1* locus.

A mitochondrial biogenesis biosensor (MitoTimer) and a doxycycline-dependent transactivator (M2rtTA) were introduced into the *AAVS1* locus of the iPSC line PD57-7 (ICGi0 52-B) with the pathogenic genetic variant c.2013T > G of the *MAPT* gene, and a healthy control (K7-4Lf, ICGi022-A) using CRISPR/Cas9 technology. Following the selection of the transfected iPSCs with antibiotics geneticin and puromycin, 40 and 58 clones, respectively, were obtained. The cells were then analysed using PCR to confirm the presence of transgenes at the *AAVS1* locus and the absence of non-specific integration of donor plasmids. After this screening, four transgenic clones from the patient iPSC and three clones from a healthy donor iPSC were chosen (Figure 2).

**Figure 3:**
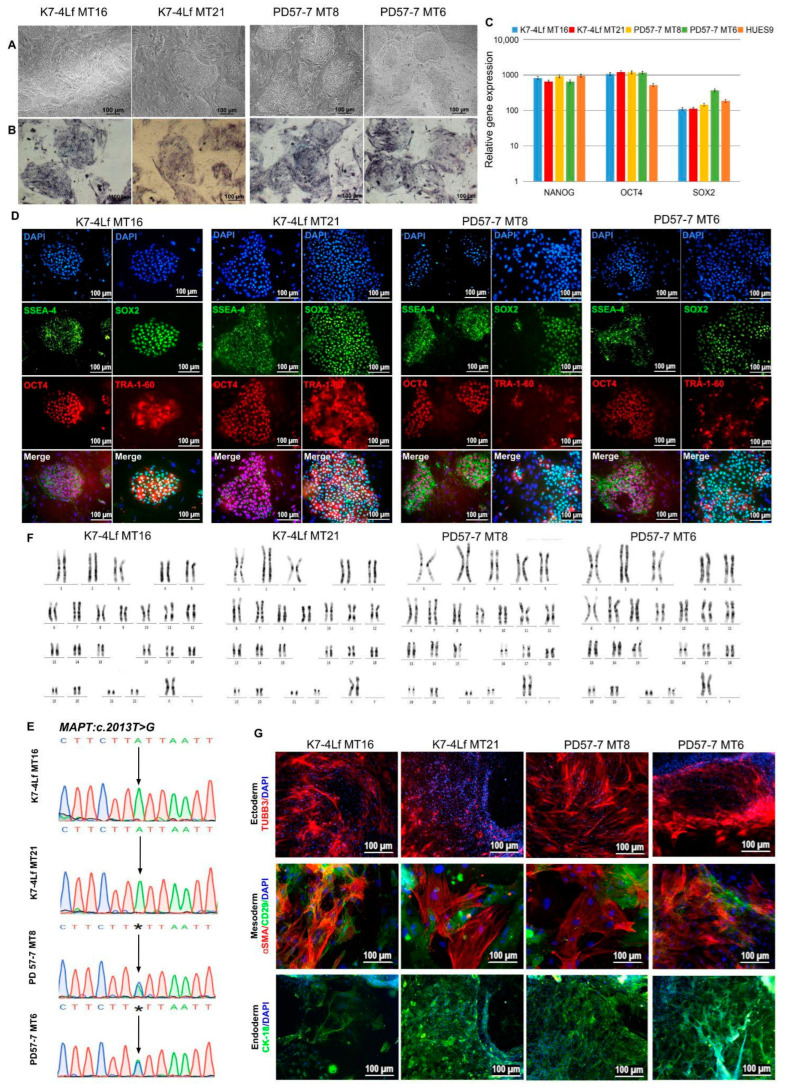
Characteristics of iPSC lines PD57-7 MT6, PD57-7 MT8, K7-4Lf MT21, and K7-4Lf MT16. (**A**) Typical morphology of iPSC colonies. (**B**) iPSCs show expression of the early pluripotency marker alkaline phosphatase. (**C**) Real-time PCR demonstrates expression of pluripotency genes *OCT4*, *SOX2*, and *NANOG*. The results were normalised to beta-2-microglobulin (*B2M)*. The standard deviation is shown using error columns. The HUES9 embryonic stem cell line was used as a positive control. (**D**) Immunofluorescence analysis shows the expression of pluripotency markers: the transcription factors OCT4 (red) and SOX2 (green), and the surface markers SSEA-4 (green) and TRA-1-60 (red). (**E**) Sequenograms of the *MAPT:c.2013T > G* gene region (indicated by arrows) of transgenic iPSC lines carrying the MitoTimer biosensor. Two iPSC lines were derived from a patient with FTDP-17 (PD57-7 MT6, PD57-7 MT8), and two iPSC lines were derived from a healthy individual (K7-4Lf MT21, K7-4Lf MT16). Asterisks (*) indicate positions of MAPT mutation (A/C). The reverse chain sequence is shown. (**F**) All four transgenic iPSC lines have a normal karyotype (46,XX). (**G**) Spontaneous differentiation into embryoid bodies and further immunofluorescence analysis of all four iPSC lines (PD57-7 MT6, PD57-7 MT8, K7-4Lf MT21, and K7-4Lf MT16) revealed the presence of three germ layers: ectoderm (TUBB3 (red)), mesoderm (αSMA (red) and CD29 (green)), and endoderm (cytokeratin 18/CK-18 (green)). Cell nuclei were stained with DAPI (blue). All scale bars are 100 µm.

Karyotyping was the first test performed for the transgenic iPSC subclones, since their generation involved genome editing. To analyse the karyotype of the iPSC lines obtained, 50 to 62 metaphase plates were examined. Chromosome segregation was performed from 6 to 12 metaphases, in accordance with the recommendations based on the European Standards for cytogenetic and molecular cytogenetic studies of constitutive and acquired chromosomal abnormalities [35]. The clones PD57-7 MT6 and PD57-7 MT8 (from the patient) and K7-4Lf MT21 and K7-4Lf MT16 (from the control) exhibited a normal karyotype of 46,XX for more than 60% of the analysed metaphases (see Figure 3F). Table 2 provides detailed information on the metaphase analysis for each iPSC line.

The morphology of the clones is typical of iPSCs (Figure 3A), the cells grow in dense monolayer colonies with a clear edge; the cultures show alkaline phosphatase activity (Figure 3B); the expression of pluripotency markers was demonstrated by qPCR (Figure 3C). Immunofluorescence analysis for specific pluripotency markers showed the expression of markers such as OCT4, SOX2, SSEA-4, TRA-1-60 (Figure 3D). Sanger sequencing confirmed the presence of the *MAPT:c.2013T > G* mutation in the patient’s transgenic clones and the absence of this substitution in the healthy control clones (Figure 3E). Immunofluorescence staining for three germ layer markers of spontaneously differentiated iPSC lines revealed derivatives of mesoderm (αSMA and CD29), ectoderm (TUBB3) and endoderm (cytokeratin 18/CK-18) (Figure 3G).

The iPSC lines were regularly tested for mycoplasma using PCR. The result of the PCR test is shown in Figure S1, which revealed no infection with this pathogen.

The obtained transgene subclones are presented in the Human Pluripotent Stem Cell Registry (https://hpscreg.eu) under the assigned names ICGi052-B-1 (PD57-7 MT8, https://hpscreg.eu/cell-line/ICGi052-B-1); ICGi052-B-2 (PD57-7 MT6, https://hpscreg.eu/cell-line/ICGi052-B-2); ICGi022-A-9 (K7-4Lf MT21, https://hpscreg.eu/cell-line/ICGi022-A-9); ICGi022-A-10 (K7-4Lf MT16, https://hpscreg.eu/cell-line/ICGi022-A-10, all web pages last accessed 28 December 2024).

### 3.3. Derivation of Dopaminergic Neurons from iPSCs

**Figure 4:**
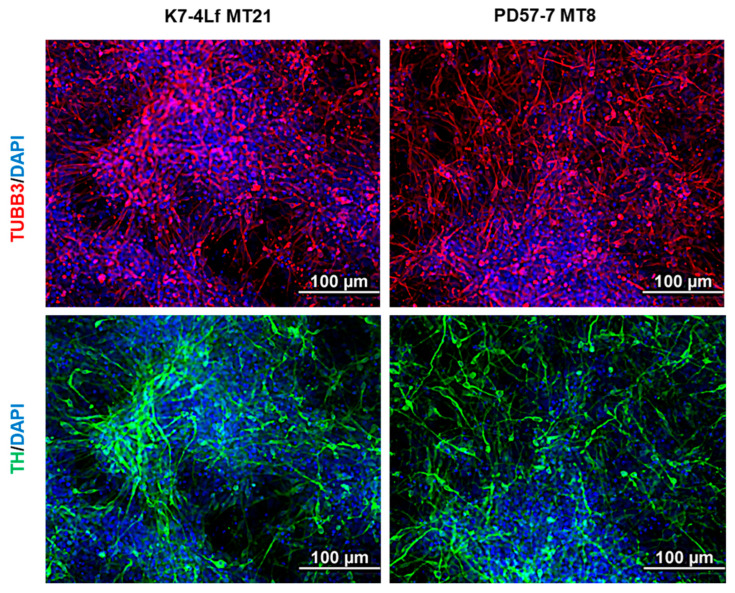
The neural derivatives of transgenic cells carrying the MitoTimer biosensor express the neural marker tubulin-beta III (TUBB3, red) and the dopaminergic neuron marker tyrosine hydroxylase (TH, green). Immunofluorescence staining was performed at days 60–75 of directed neuronal differentiation. Nuclei were counterstained with DAPI (blue). All scale bars are 100 µm.

To validate the MitoTimer readout in the relevant cell type, transgenic iPSC clones were differentiated into dopaminergic neurons. All differentiated cell cultures showed the presence of TUBB3, the major neuronal marker, as well as the marker of mature dopaminergic neurons—tyrosine hydroxylase/TH—at days 60–75 of neuronal differentiation (Figure 4).

### 3.4. The Investigation of MitoTimer Biosensor Readout in Transgenic iPSC-Derived Neurons

The neural derivatives of transgenic iPSC lines PD57-7 MT6, PD57-7 MT8, K7-4Lf MT21, and K7-4Lf MT16 were cultured for two days in the presence of doxycycline to activate the expression of the MitoTimer biosensor. MitoTimer is a fluorescent protein that is delivered to the mitochondria and emits a green light when a new protein is synthesised. However, the protein maturation leads to the peak of the fluorescence emission shifting irreversibly to the red, corresponding to its oxidation and “aging” [15]. We demonstrated the time-dependent triggering of the MitoTimer in neuronal cells after the addition of doxycycline (Figure S2). In transgenic lines, the appearance of the green MitoTimer in mitochondria is observed shortly after the addition of doxycycline, as the addition of doxycycline leads to the formation of a complex with the constitutively expressed transactivator and activates the MitoTimer transgene [36]. Subsequent to the induction of MitoTimer expression, an augmentation in the green signal is observed within a two-hour time frame. This is followed by the conversion of green protein to red, which subsequently remains constant during the 23 h observation period on the Cell-IQ instrument. This observation is consistent with the existing literature [16].

**Figure 5:**
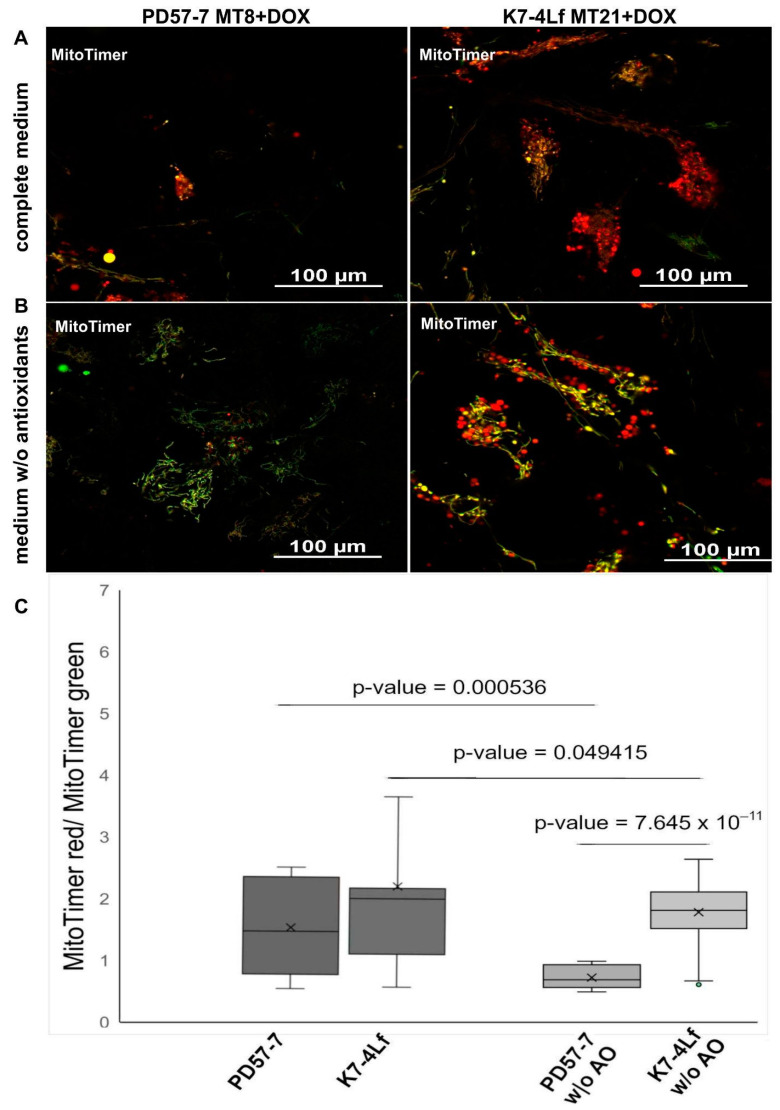
MitoTimer distribution pattern in neuronal derivatives of transgenic iPSC clones. (**A**) Fluorescent signals of the MitoTimer biosensor in the PD57-7 MT8 and K7-4Lf MT21 neural derivatives growing in complete medium. Green signals correspond to the newly synthesised MitoTimer protein, red signals are aged MitoTimer protein, and yellow signals correspond to the colocalisations of green (new) and red (old) MitoTimer. (**B**) Fluorescent signals of the MitoTimer biosensor in the PD57-7 MT8 and K7-4Lf MT21 neural derivatives after two days of antioxidant-free cultivation. All scale bars are 100 µm. (**C**) A comparison of the ratio of the red/green MitoTimer signal in neural derivatives in a complete medium and medium without antioxidants. An ANOVA test was utilised to estimate *p*-values.

The MitoTimer readout was calculated in neuronal cells growing in both full medium and an antioxidant-depleted medium (Figure 5C). The red/green ratio in K7-4Lf-MT and PD57-MT neurons that were cultured on complete medium in the presence of doxycycline was comparable. However, in an antioxidant-depleted medium, significant differences are observed between the patient’s cells and the control. The average value of the ratio significantly decreased when neurons were cultured in the absence of antioxidants (from 1.49 to 0.72 in the patient’s cells and from 2.17 to 1.78 in the control).

## 4. Discussion

Neurodegenerative diseases are characterised by the accumulation of proteins such as tau and a-synuclein. Proteinopathies develop in parallel with metabolic disorders such as impaired tricarboxylic acid cycle function, deficient oxidative phosphorylation, and reduced energy support to neurons, which exacerbate mitochondrial dysfunction [37,38]. Research into mitochondrial dysfunction is essential for finding ways to combat the processes that lead to malfunction and death in different types of cells. Mitochondria are responsible for the synthesis of ATP owing to three protein complexes located in the inner mitochondrial membrane. These complexes are part of the respiratory electron transport chain and are involved in oxidative phosphorylation. However, as tauopathies develop, tau protein binds to mitochondrial cardiolipin, thereby blocking the function of respiratory chain complexes. Furthermore, when tau protein is overexpressed, it is able to bind to parkin, blocking mitophagy and contributing to the accumulation of damaged (fragmented/inactive) mitochondria. This contributes to the development of mitochondrial dysfunction, since the blocking of mitophagy also reduces the synthesis of new, intact mitochondria [39,40]. The study of mitochondrial function in the development of tauopathies may contribute to the discovery of the most effective way to restore mitochondrial biogenesis.

Models based on neurons derived from iPSCs of patients with different genetic variants of the *MAPT* gene show a wide range of pathogenetic changes that include accumulation of reactive oxygen species, exudative stress, impaired mitochondrial bioenergetics, and pathologic changes in the proteostasis system, including lysosome dysfunction, dysfunction of intracellular microvesicular trafficking, and calcium homeostasis [7,9,12,41,42,43,44]. At the same time, most research methods, such as gene expression studies using quantitative PCR, bulk RNA-Seq, Western blot, and immunocytochemical analysis, require cell fixation and lysis. However, the use of the genetically encoded biosensor MitoTimer allows us to study the dynamics of mitochondrial biogenesis and turnover in vivo. This provides additional opportunities to obtain data, for example, following exposure to potentially toxic or medicinal chemical compounds or investigational gene therapy agents [15,17,18,19,20,21]. The transgenesis scheme utilised in this study, in contrast to the use of temporary plasmid transfection and the employment of lentiviral vectors, facilitates the precise control of the number of transgenes per genome. This is a crucial aspect in the comparative analysis of experimental and control lines, as well as cell clones. Furthermore, the doxycycline-activated expression of MitoTimer, utilised in our approach, facilitates biosensor triggering at various stages of differentiation of iPSCs into specific cell types, including dopaminergic neurons. Additionally, pulse triggering of MitoTimer protein expression enables the study of mitochondrial turnover [21]. The model’s foundation on pluripotent cells enables the study of mitochondrial biogenesis and turnover not only in dopaminergic neurons but also in other cell types with the same genotype. This provides novel fundamental data on the peculiarities of these processes depending on the cellular context and new opportunities for using this model to test promising drugs on a wider range of cell types. This is of particular importance when evaluating the side effects of substances at the preclinical stages of drug development [45,46].

As a model to study mitochondrial dysfunction, we selected neuronal cells derived from iPSCs of a patient with frontotemporal dementia with parkinsonism-17 carrying the pathogenic mutation c.2013T > G (p.N279K) in the *MAPT* gene. The genetic variant of *MAPT* is a missense mutation whose primary effects are manifested at the mRNA level [5]. The frequency of inclusion of exon 10 in the transcript increases during splicing [8]. As a result, the ratio of tau protein 3R and 4R forms changes towards an increase in the level of the latter, leading to destabilisation of microtubules and disruption of subcellular vesicle transport. Increased calcium fluctuations were observed in MAPT p.N279K neurons, leading to hyperpolarisation and mitochondrial fission. This led to an increase in mitochondrial ROS production and further cell death [9].

To facilitate the lifetime visualisation of mitochondrial biogenesis, genetically encoded biosensors are employed. The natural oxidising ability of Timer protein during cell life was utilised to create a genetic construct encoding MitoTimer for doxycycline-dependent expression [17]. In this work, we have created a cellular model to study mitochondrial biogenesis in frontotemporal dementia with parkinsonism-17 based on iPSC-derived neuronal cells. Using CRISPR-Cas9 technology, we obtained transgenic iPSC lines of patient PD57-7 and healthy control K7-4Lf. These lines contain genetic constructs for the expression of the MitoTimer biosensor and doxycycline-dependent reverse transactivator at the *AAVS1* locus. The *AAVS1* locus is located in the *PPP1R12C* gene on chromosome 19 and is characterised by an open chromatin structure surrounded by insulating elements. These elements prevent transactivation or repression of inserted genes [47]. Transgenes integrated at this locus show stable transcriptional activity both in iPSCs and during their differentiation into other cell types [48]. Disruptions in *PPP1R12C* are not associated with known diseases, so the *AAVS1* locus is often considered a safe zone for transgene integration [49]. Integration of the transgene into the *AAVS1* locus allows, in contrast to the use of lentiviral vectors or transient transfection of plasmid DNA, control of the number of transgene copies per genome, which reduces the possible toxic effect of its overexpression. In addition, this approach allows more reliable comparison of signal intensity and their ratio when comparing patient-specific and control cell lines and clones, which is especially important when using fluorescent ratiometric biosensors.

The employment of an inducible promoter (for instance, one controlled by doxycycline) ensures regulated expression, thereby minimising deleterious side and non-target effects [47,48,49,50,51,52]. After neuronal differentiation of the transgenic iPSC lines, the MitoTimer biosensor was activated with doxycycline. Transgene expression was observed in neuronal derivatives obtained from transgenic clones of both the patient and the control.

Although the ratio of MitoTimer signals in the neuronal derivatives of the patient was compared to that of the control in the complete medium. However, in neurons from the FTDP-17 patient, cells have been observed in which greener and redder mitochondria have been detected. By contrast, in neurons from the healthy control, mitochondria are predominantly observed in which the green and red MitoTimer signal is colocalised (visualised as yellow). This may indicate a violation of the fusion and fission process in PD57-7 MT8, which contributes to the “homogenisation” of new and ageing MitoTimer. Cultivation of K7-4Lf MT neurons in a medium without antioxidants in the presence of doxycycline resulted in a slight significant decrease in the MitoTimer red/green ratio, whereas for PD57-7 MT8, this decrease was dramatic and shifted towards green (Figure 5C).

Numerous studies have demonstrated that the capacity to shift the green-to-red MitoTimer ratio is exclusive to mitochondrial biogenesis and degradation processes. It is noteworthy that the process of oxidative stress itself exerts no influence on the green-to-red MitoTimer transformation. In neurodegenerative diseases, mitochondria are subject to a multitude of influences, including oxidative stress. In the absence of T8 iPSCs, is likely explained by a fusion disruption that prevents the mixing of the old and new MitoTimer protein in the mitochondrial population, combined with a reduced ability to import a new protein into the “old” mitochondria [16]. Interestingly, in our study, we showed the elimination of the aged MitoTimer in FTDP-17 patient neurons when cultured in a medium without antioxidants. With a constant rate of green protein formation and its conversion to red, a shift in the ratio is possible with mitochondrial degradation. According to the literature data, mitophagy processes are disrupted in tauopathy [8], which should lead to the accumulation of red ageing MitoTimer, but in our experiments we observe the elimination of ageing MitoTimer, most likely together with defective mitochondria. This finding requires further investigation.

The present study provides a functional cellular system for the in vitro investigation of factors that affect mitochondrial turnover. In the future, this model can be used to screen for drugs that alter mitochondrial biogenesis.

## 5. Conclusions

In this study, for the first time, iPSCs carrying the p.N279K genetic variant of the *MAPT* gene were utilised to create a test model for studying mitochondrial biogenesis alterations caused by tauopathy. This was achieved using the genetically encoded MitoTimer biosensor, which was developed for imaging mitochondrial turnover. The employment of iPSCs bearing the MitoTimer biosensor facilitates in vitro investigation of mitochondrial function across diverse cell types, thereby enabling the screening of drugs designed to modulate mitochondrial activity. It is acknowledged that the model developed is presently constrained to the c.2013T > G variant of the *MAPT* gene. To expand the scope of the model, it is necessary to extend the range of transgenic lines utilised to include other variants of *MAPT*, as well as pathogenic variants of other genes associated with frontotemporal dementia and parkinsonism.

## Figures and Tables

**Table 1:** Sequences of primers used in the work.

	Target	Size of Band	Forward/Reverse Primer (5′–3′)
Housekeeping gene (RT-qPCR)	B2M	90 bp	TAGCTGTGCTCGCGCTACT/ TCTCTGCTGGATGACGTGAG
Pluripotency marker (RT-qPCR)	*NANOG*	116 bp	TTTGTGGGCCTGAAGAAAACT/ AGGGCTGTCCTGAATAAGCAG
	*OCT4*	94 bp	CTTCTGCTTCAGGAGCTTGG/ GAAGGAGAAGCTGGAGCAAA
	*SOX2*	100 bp	GCTTAGCCTCGTCGATGAAC/ AACCCCAAGATGCACAACTC
Mycoplasma detection	16S ribosomal RNA gen	280 bp	GGGAGCAAACAGGATTAGATACCCT/ TGCACCATCTGTCACTCTGTTAACCTC
*MAPT* mutation analysis	*MAPT:c.2013T > G *	427 bp	TCGTAAAGCCCGCTGGAAAT/ GTGTACGCACTCACACCACT
Expression of 3R and 4R transcript form *MAPT*	3R and 4R *MAPT*	305 bp (3R) 398 bp (4R)	GTCAAGTCCAAGATCGGCTC/ TGGTCTGTCTTGGCTTTGGC
Detection of the wild-type *AAVS1* allele	*AAVS1*	555 bp	CTCTGGCTCCATCGTAAGCAA/ CCCAAAGTACCCCGTCTCCC
Integration of the *M2rtTA* transgene into the *AAVS1* locus	*Neo-M2rtTA*	1024 bp	CCGGACCACTTTGAGCTCTAC/ GCCCAGTCATAGCCGAATAG
Integration of the MitoTimer transgene into the *AAVS1* locus	*Puro-TRE-mCMV-MitoTimer*	1022 bp	CCGGACCACTTTGAGCTCTAC/ AGGCGCACCGTGGGCTTGTAC
Off-target integration of the AAVS1-Neo- M2rtTAplasmid into the genome	*AAVS1-Neo-M2rtTA*	1063 bp	CAGGAAACAGCTATGAC/ GCCCAGTCATAGCCGAATAG
Off-target integration of the pAAVS1-TRE-mCMV-MitoTimer plasmid into the genome	*pAAVS1-TRE-mCMV-MitoTimer*	1007 bp	CAGGAAACAGCTATGAC/ GCCCAGTCATAGCCGAATAG

**Table 2:** Detailed analysis of the chromosomal composition of metaphase plates of the obtained iPSC lines.

Cell Line	% of Polyploid Metaphases	% of Metaphases with Different Number of Chromosomes					Number of Analysed Metaphases	Karyotypes in Diploid Cells
		42–44	45	46	47	48		
K7-4Lf MT16	5.4	11	12.7	60	5.4	5.4	55	46,XX
K7-4Lf MT21	8	4	4	80	4	0	50	46,XX
PD57-7 MT6	5.3	12.5	12.5	66.1	1.8	1.8	56	46,XX
PD57-7 MT8	6.4	6.4	12.9	67.8	6.4	0	62	46,XX

## Data Availability

The characteristics of the iPSCs are presented in the manuscript and can be found in the Human Pluripotent Stem Cell Registry (hPSCreg; https://hpscreg.eu) including PD57-7/ICGi052-B (PD57-7 MT8; PD57-7 MT6, https://hpscreg.eu/cell-line/ICGi052-B-2) and healthy donor K7-4Lf/ICGi022-A (K7-4Lf MT21, https://hpscreg.eu/cell-line/ICGi022-A-9; K7-4Lf MT16, https://hpscreg.eu/cell-line/ICGi022-A-10). All web pages were last accessed on 28 December 2024.

## Supplementary Materials

The following supporting information can be downloaded at https://www.mdpi.com/article/10.3390/biomedicines13030550/s1: Table S1. Antibodies used in the work. Figure S1. PCR-test for mycoplasma contamination is negative. Figure S2. Changes in MitoTimer red/green fluorescence ratio in dopaminergic neurons differentiated from PD57 patient iPSCs in the first 23 h after addition of doxycycline to the growth medium. The trend line is shown as a dashed line.

## References

[1] Creekmore B.C., Watanabe R., Lee E.B. (2024). Neurodegenerative Disease Tauopathies. Annu. Rev. Pathol. Mech. Dis..

[2] Chu Y., Hirst W.D., Federoff H.J., Harms A.S., Stoessl A.J., Kordower J.H. (2024). Nigrostriatal tau pathology in parkinsonism and Parkinson’s disease. Brain.

[3] Espay A.J., Lees A.J. (2024). Are we entering the ‘Tau-lemaic’ era of Parkinson’s disease?. Brain.

[4] Strang K.H., Golde T.E., Giasson B.I. (2019). MAPT mutations, tauopathy, and mechanisms of neurodegeneration. Lab. Investig..

[5] Hasegawa M., Smith M.J., Iijima M., Tabira T., Goedert M. (1999). FTDP-17 mutations N279K and S305N in tau produce increased splicing of exon 10. FEBS Lett..

[6] Hong M., Zhukareva V., Vogelsberg-Ragaglia V., Wszolek Z., Reed L., Miller B.I., Geschwind D.H., Bird T.D., McKeel D., Goate A. (1998). Mutation-Specific Functional Impairments in Distinct Tau Isoforms of Hereditary FTDP-17. Science.

[7] Wren M.C., Zhao J., Liu C.-C., Murray M.E., Atagi Y., Davis M.D., Fu Y., Okano H.J., Ogaki K., Strongosky A.J. (2015). Frontotemporal dementia-associated N279K tau mutant disrupts subcellular vesicle trafficking and induces cellular stress in iPSC-derived neural stem cells. Mol. Neurodegener..

[8] Ritter M.L., Avila J., García-Escudero V., Hernández F., Pérez M. (2018). Frontotemporal Dementia-Associated N279K Tau Mutation Localizes at the Nuclear Compartment. Front. Cell. Neurosci..

[9] Korn L., Speicher A.M., Schroeter C.B., Gola L., Kaehne T., Engler A., Disse P., Fernández-Orth J., Csatári J., Naumann M. (2023). *MAPT* genotype-dependent mitochondrial aberration and ROS production trigger dysfunction and death in cortical neurons of patients with hereditary FTLD. Redox Biol..

[10] Szabo L., Grimm A., García-León J.A., Verfaillie C.M., Eckert A. (2023). Genetically Engineered Triple MAPT-Mutant Human-Induced Pluripotent Stem Cells (N279K, P301L, and E10+16 Mutations) Exhibit Impairments in Mitochondrial Bioenergetics and Dynamics. Cells.

[11] Rodriguez-Martin T., Anthony K., Garcia-Blanco M.A., Mansfield S.G., Anderton B.H., Gallo J.-M. (2009). Correction of tau mis-splicing caused by FTDP-17 MAPT mutations by spliceosome-mediated RNA trans-splicing. Hum. Mol. Genet..

[12] Iovino M., Agathou S., González-Rueda A., Del Castillo Velasco-Herrera M., Borroni B., Alberici A., Lynch T., O’Dowd S., Geti I., Gaffney D. (2015). Early maturation and distinct tau pathology in induced pluripotent stem cell-derived neurons from patients with MAPT mutations. Brain.

[13] Valetdinova K.R., Malankhanova T.B., Zakian S.M., Medvedev S.P. (2021). The Cutting Edge of Disease Modeling: Synergy of Induced Pluripotent Stem Cell Technology and Genetically Encoded Biosensors. Biomedicines.

[14] Hernandez G., Thornton C., Stotland A., Lui D., Sin J., Ramil J., Magee N., Andres A., Quarato G., Carreira R.S. (2013). MitoTimer: A novel tool for monitoring mitochondrial turnover. Autophagy.

[15] Ferree A.W., Trudeau K., Zik E., Benador I.Y., Twig G., Gottlieb R.A., Shirihai O.S. (2013). MitoTimer probe reveals the impact of autophagy, fusion, and motility on subcellular distribution of young and old mitochondrial protein and on relative mitochondrial protein age. Autophagy.

[16] Trudeau K.M., Gottlieb R.A., Shirihai O.S. (2014). Measurement of mitochondrial turnover and life cycle using MitoTimer. Methods in Enzymology.

[17] Laker R.C., Xu P., Ryall K.A., Sujkowski A., Kenwood B.M., Chain K.H., Zhang M., Royal M.A., Hoehn K.L., Driscoll M. (2014). A Novel *MitoTimer* Reporter Gene for Mitochondrial Content, Structure, Stress, and Damage in Vivo. J. Biol. Chem..

[18] Stotland A., Gottlieb R.A. (2016). α-MHC MitoTimer mouse: In vivo mitochondrial turnover model reveals remarkable mitochondrial heterogeneity in the heart. J. Mol. Cell. Cardiol..

[19] Wilson R.J., Drake J.C., Cui D., Zhang M., Perry H.M., Kashatus J.A., Kusminski C.M., Scherer P.E., Kashatus D.F., Okusa M.D. (2019). Conditional MitoTimer reporter mice for assessment of mitochondrial structure, oxidative stress, and mitophagy. Mitochondrion.

[20] Ma X., Ding W.-X., Papa S., Bubici C. (2023). Methods for Monitoring Mitochondrial Biogenesis and Turnover in Cultured Hepatocytes and Mouse Liver Using MitoTimer Reporter Assay. Metabolic Reprogramming: Methods and Protocols.

[21] Gottlieb R.A., Stotland A. (2015). MitoTimer: A novel protein for monitoring mitochondrial turnover in the heart. J. Mol. Med..

[22] Cerqueira F., Kozer N., Petcherski A., Baranovski B. (2020). MitoTimer-based high-content screen identifies two chemically-related benzothiophene derivatives that enhance basal mitophagy. Biochem. J..

[23] Epremyan K.K., Goleva T.N., Zvyagilskaya R.A. (2022). Effect of Tau Protein on Mitochondrial Functions. Biochem. Mosc..

[24] Cheng Y., Bai F. (2018). The Association of Tau With Mitochondrial Dysfunction in Alzheimer’s Disease. Front. Neurosci..

[25] Grigor’eva E.V., Malakhova A.A., Yarkova E.S., Minina J.M., Vyatkin Y.V., Nadtochy J.A., Khabarova E.A., Rzaev J.A., Medvedev S.P., Zakian S.M. (2024). Generation and characterization of two induced pluripotent stem cell lines (ICGi052-A and ICGi052-B) from a patient with frontotemporal dementia with parkinsonism-17 associated with the pathological variant c.2013T>G in the MAPT gene. Vavilovskii Zhurnal Genet. I Sel..

[26] Malakhova A.A., Grigor’eva E.V., Pavlova S.V., Malankhanova T.B., Valetdinova K.R., Vyatkin Y.V., Khabarova E., Rzaev J., Zakian S., Medvedev S. (2020). Generation of induced pluripotent stem cell lines ICGi021-A and ICGi022-A from peripheral blood mononuclear cells of two healthy individuals from Siberian population. Stem Cell Res..

[27] Ustyantseva E., Pavlova S.V., Malakhova A.A., Ustyantsev K., Zakian S.M., Medvedev S.P. (2022). Oxidative stress monitoring in iPSC-derived motor neurons using genetically encoded biosensors of H_2_O_2_. Sci. Rep..

[28] Ran F.A., Hsu P.D., Wright J., Agarwala V., Scott D.A., Zhang F. (2013). Genome engineering using the CRISPR-Cas9 system. Nat. Protoc..

[29] DeKelver R.C., Choi V.M., Moehle E.A., Paschon D.E., Hockemeyer D., Meijsing S.H., Sancak Y., Cui X., Steine E.J., Miller J.C. (2010). Functional genomics, proteomics, and regulatory DNA analysis in isogenic settings using zinc finger nuclease-driven transgenesis into a safe harbor locus in the human genome. Genome Res..

[30] Yarkova E.S., Grigor’eva E.V., Medvedev S.P., Tarasevich D.A., Pavlova S.V., Valetdinova K.R., Minina J.M., Zakian S.M., Malakhova A.A. (2024). Detection of ER stress in iPSC-derived neurons carrying the p.N370S mutation in the *GBA1* gene. Biomedicines.

[31] Grigor’eva E.V., Kopytova A.E., Yarkova E.S., Pavlova S.V., Sorogina D.A., Malakhova A.A., Malankhanova T.B., Baydakova G.V., Zakharova E.Y., Medvedev S.P. (2023). Biochemical Characteristics of iPSC-Derived Dopaminergic Neurons from N370S GBA Variant Carriers with and without Parkinson’s Disease. Int. J. Mol. Sci..

[32] Oosterveen T., Garção P., Moles-Garcia E., Soleilhavoup C., Travaglio M., Sheraz S., Peltrini R., Patrick K., Labas V., Combes-Soia L. (2021). Pluripotent stem cell derived dopaminergic subpopulations model the selective neuron degeneration in Parkinson’s disease. Stem Cell Rep..

[33] Cowan C.A., Klimanskaya I., McMahon J., Atienza J., Witmyer J., Zucker J.P., Wang S., Morton C.C., McMahon A.P., Powers D. (2004). Derivation of embryonic stem-cell lines from human blastocysts. N. Engl. J. Med..

[34] Livak K.J., Schmittgen T.D. (2001). Analysis of Relative Gene Expression Data Using Real-Time Quantitative PCR and the 2−ΔΔCT Method. Methods.

[35] McGowan-Jordan J., Hastings R.J., Moore S. (2020). ISCN 2020: An International System for Human Cytogenomic Nomenclature. Cytogenetic and Genome Research.

[36] Gossen M., Freundlieb S., Bender G., Müller G., Hillen W., Bujard H. (1995). Transcriptional Activation by Tetracyclines in Mammalian Cells. Science.

[37] Murphy M.P., Hartley R.C. (2018). Mitochondria as a therapeutic target for common pathologies. Nat. Rev. Drug Discov..

[38] Picard M., Shirihai O.S. (2022). Mitochondrial signal transduction. Cell Metab..

[39] Ardail D., Privat J.P., Egret-Charlier M., Levrat C., Lerme F., Louisot P. (1990). Mitochondrial contact sites. Lipid composition and dynamics. J. Biol. Chem..

[40] Paradies G., Paradies V., De Benedictis V., Ruggiero F.M., Petrosillo G. (2014). Functional role of cardiolipin in mitochondrial bioenergetics. Biochim. Biophys. Acta Bioenerg..

[41] Esteras N., Rohrer J.D., Hardy J., Wray S., Abramov A.Y. (2017). Mitochondrial hyperpolarization in iPSC-derived neurons from patients of FTDP-17 with 10+16 *MAPT* mutation leads to oxidative stress and neurodegeneration. Redox Biol..

[42] Verheyen A., Diels A., Reumers J., Van Hoorde K., Van den Wyngaert I., van Outryve d’Ydewalle C., De Bondt A., Kuijlaars J., De Muynck L., De Hoogt R. (2018). Genetically Engineered iPSC-Derived FTDP-17 *MAPT* Neurons Display Mutation-Specific Neurodegenerative and Neurodevelopmental Phenotypes. Stem Cell Rep..

[43] Minaya M.A., Mahali S., Iyer A.K., Eteleeb A.M., Martinez R., Huang G., Budde J., Temple S., Nana A.L., Seeley W.W. (2023). Conserved gene signatures shared among MAPT mutations reveal defects in calcium signaling. Front. Mol. Biosci..

[44] Mahali S., Martinez R., King M., Verbeck A., Harari O., Benitez B.A., Horie K., Sato C., Temple S., Karch C.M. (2022). Defective proteostasis in induced pluripotent stem cell models of frontotemporal lobar degeneration. Transl. Psychiatry.

[45] Elitt M.S., Barbar L., Tesar P.J. (2018). Drug screening for human genetic diseases using iPSC models. Hum. Mol. Genet..

[46] Rowe R.G., Daley G.Q. (2019). Induced pluripotent stem cells in disease modelling and drug discovery. Nat. Rev. Genet..

[47] Merling R.K., Sweeney C.L., Chu J., Bodansky A., Choi U., Priel D.L., Kuhns D.B., Wang H., Vasilevsky S., De Ravin S.S. (2015). An AAVS1-Targeted Minigene Platform for Correction of iPSCs From All Five Types of Chronic Granulomatous Disease. Mol. Ther..

[48] Bharucha N., Ataam J.A., Gavidia A.A., Karakikes I. (2021). Generation of AAVS1 integrated doxycycline-inducible CRISPR-Prime Editor human induced pluripotent stem cell line. Stem Cell Res..

[49] Oceguera-Yanez F., Kim S.-I., Matsumoto T., Tan G.W., Xiang L., Hatani T., Kondo T., Ikeya M., Yoshida Y., Inoue H. (2016). Engineering the AAVS1 locus for consistent and scalable transgene expression in human iPSCs and their differentiated derivatives. Methods.

[50] Tiyaboonchai A., Mac H., Shamsedeen R., Mills J.A., Kishore S., French D.L., Gadue P. (2014). Utilization of the AAVS1 safe harbor locus for hematopoietic specific transgene expression and gene knockdown in human ES cells. Stem Cell Res..

[51] Sadelain M., Papapetrou E.P., Bushman F.D. (2011). Safe harbours for the integration of new DNA in the human genome. Nat. Rev. Cancer.

[52] Hayashi H., Kubo Y., Izumida M., Matsuyama T. (2020). Efficient viral delivery of Cas9 into human safe harbor. Sci. Rep..

