# Supplementary material for: Transgenic iPSC Lines with Genetically Encoded MitoTimer to Study Mitochondrial Biogenesis in Dopaminergic Neurons with Tauopathy
Source: Biomedicines. 2025 Feb 21;13(3):550. doi: 10.3390/biomedicines13030550 (PMC11940372; doi:10.3390/biomedicines13030550)
Supplement: Supplementary file 1 [file biomedicines-13-00550-s001.zip › biomedicines-3429884-supplementary.pdf]

Supplementary materials

Table S1. Antibodies used in the work

**Antibody**	**Dilution**	**Company Cat.**	**RRID**
Pluripotency markers			
Rabbit IgG anti-OCT4	1:200	Abcam, ab18976	RRID:AB_444714
Mouse IgM anti-TRA-1-60		AbcamCat.#ab16288	RRID:AB_778563
Mouse IgG3 anti-SSEA-4		Abcam, ab16287	RRID:AB_778073
Rabbit IgG anti-SOX2	1:500	CellSignalingCat.#3579	RRID:AB_2195767
Differentiation markers			
Mouse IgG2a anti-αSMA	1:200	Dako, M0851	RRID:AB_2223500
Mouse IgG1 anti-CD29 (Integrin beta 1) (TS2/16)	1:100	Thermo Fisher Scientific, 14-0299-82,	RRID:AB_1210468
Mouse IgG2a anti-Tubulin β 3 (TUBB3)/ Clone: TUJ1	1:1000	BioLegend, 801201	RRID:AB_2313773
Mouse IgG1 anti-Cytokeratin 18 (KRT18)	1:250	Abcam, ab668	RRID:AB_305647
Mouse IgG1 anti- HNF3β/FOXA2	1:50	Santa Cruz Biotechnology, sc-374376	RRID:AB_10989742
Secondary antibodies			
Goat anti-Mouse IgG1 Alexa Fluor 568	1:400	Thermo Fisher Scientific, A21124	RRID:AB_2535766
Goat anti-mouse IgG1 Alexa Fluor 488		Thermo Fisher Scientific, A21121	RRID:AB_2535764
Goat anti-Mouse IgG2a Cross-Adsorbed Secondary Antibody, Alexa Fluor 568		Thermo Fisher Scientific, A21134	RRID:AB_2535773
Goat anti-Mouse IgG2a Alexa Fluor 488		Thermo Fisher Scientific, A21131	RRID:AB_2535771
Goat anti-Mouse IgG3 Cross-Adsorbed Secondary Antibody, Alexa Fluor 488		Thermo Fisher Scientific, A21151	RRID:AB_2535784
Goat anti-Mouse IgM Heavy Chain Cross-Adsorbed Secondary Antibody, Alexa Fluor 568		Thermo Fisher Scientific, A21043	RRID:AB_2535712
Goat anti-Rabbit IgG (H + L) Highly Cross-Adsorbed Secondary Antibody, Alexa Fluor 488		Thermo Fisher Scientific, A11008	RRID:AB_143165
Goat anti-Rabbit IgG (H + L) Secondary Antibody, Alexa Fluor 568		Thermo Fisher Scientific, A11011	RRID:AB_143157

Test for mycoplasma contamination

The iPSC lines were free of mycoplasma contamination.

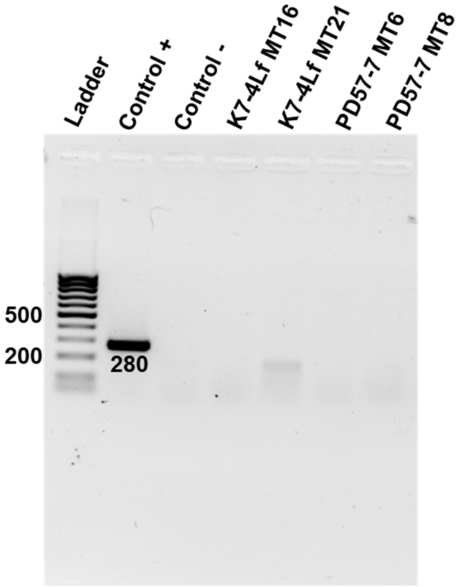

Figure S1. PCR-test for mycoplasma contamination is negative.

Time-dependent changes in the red/green ratio of the MitoTimer biosensor

The alterations in the red/green ratio of the MitoTimer biosensor fluorescent signals in iPSC-derived neurons after the addition of doxycycline to the growth medium were estimated. The fluorescent signals were monitored using the Cell-IQ real-time cell analyser (CM Technologies Oy) at the Cell Technology Facility of the Institute of Cytology and Genetics SB RAS.

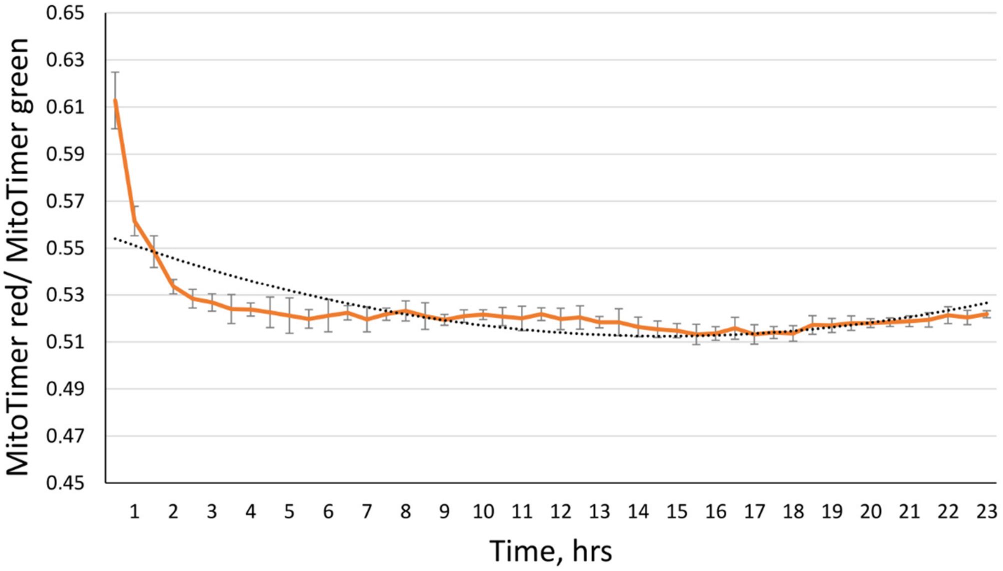

Figure S2. Changes in MitoTimer red/green fluorescence ratio in dopaminergic neurons differentiated from PD57 patient iPSCs in the first 23 hours after addition of doxycycline to the growth medium. The trend line is shown as a dashed line.
